# Hookah smoking and cancer: carcinoembryonic antigen (CEA) levels in exclusive/ever hookah smokers

**DOI:** 10.1186/1477-7517-5-19

**Published:** 2008-05-24

**Authors:** Khan Mohammad Sajid, Kamal Chaouachi, Rubaida Mahmood

**Affiliations:** 1 Multan Institute of Nuclear Medicine and Radiotherapy (MINAR), Multan, Pakistan; 2 Paris XI University (Lecturer DIU Tabacologie/Narghile), Paris Sud, France; 3 MINAR (Scientific Collaborator), Multan, Pakistan

## Abstract

**Background:**

We have recently published some work on CEA levels in hookah (also called narghile, shisha elsewhere) and cigarette smokers. Hookah smokers had higher levels of CEA than non-smokers although mean levels were low compared to cigarette smokers. However some of them were also users of other tobacco products (cigarettes, bidis, etc.).

**Objectives:**

To find serum CEA levels in ever/exclusive hookah smokers, i.e. those who smoked only hookah (no cigarettes, bidis, etc.), prepared between 1 and 4 times a day with a quantity of up to 120 g of a tobacco-molasses mixture each (i.e. the tobacco weight equivalent of up to 60 cigarettes of 1 g each) and consumed in 1 to 8 sessions.

**Methods:**

Enhanced chemiluminescent immunometric technique was applied to measure CEA levels in serum samples from 59 exclusive male smokers with age ranging from 20–80 years (mean = 58.8 ± 14.7 years) and 8–65 years of smoking (mean = 37.7 ± 16.8). 36 non-smokers served as controls. Subjects were divided into 3 groups according to the number of preparations; the number of sessions and the total daily smoking time: Light (1; 1; ≤ 20 minutes); Medium (1–3; 1–3; >20 min to ≤ 2 hrs) and Heavy smokers (2–4; 3–8; >2 hrs to ≤ 6 hrs). Because of the nature of distribution of CEA levels among our individuals, Wilcoxon's rank sum two-sample test was applied to compare the variables.

**Results:**

The overall CEA levels in exclusive hookah smokers (mean: 3.58 ± 2.61 ng/ml; n = 59) were not significantly different (p ≤ 0.0937) from the levels in non-smokers (2.35 ± 0.71 ng/ml). Mean levels in light, medium and heavy smokers were: 1.06 ± 0.492 ng/ml (n = 5); 2.52 ± 1.15 ng/ml (n = 28) and 5.11 ± 3.08 ng/ml (n = 26) respectively. The levels in medium smokers and non-smokers were also not significantly different (p ≤ 0.9138). In heavy smokers, the CEA levels were significantly higher than in non-smokers (p ≤ 0.0001567).

**Conclusion:**

Overall CEA levels in exclusive hookah smokers were low compared to cigarette smokers. However, heavy hookah smoking substantially raises CEA levels. Low-nitrosamines smokeless tobacco of the SNUS Swedish type could be envisaged as an alternative to smoking for this category of users and also, in a broad harm reduction perspective, to the prevalent low-quality moist snuff called naswar.

## Background

**Figure 1:**
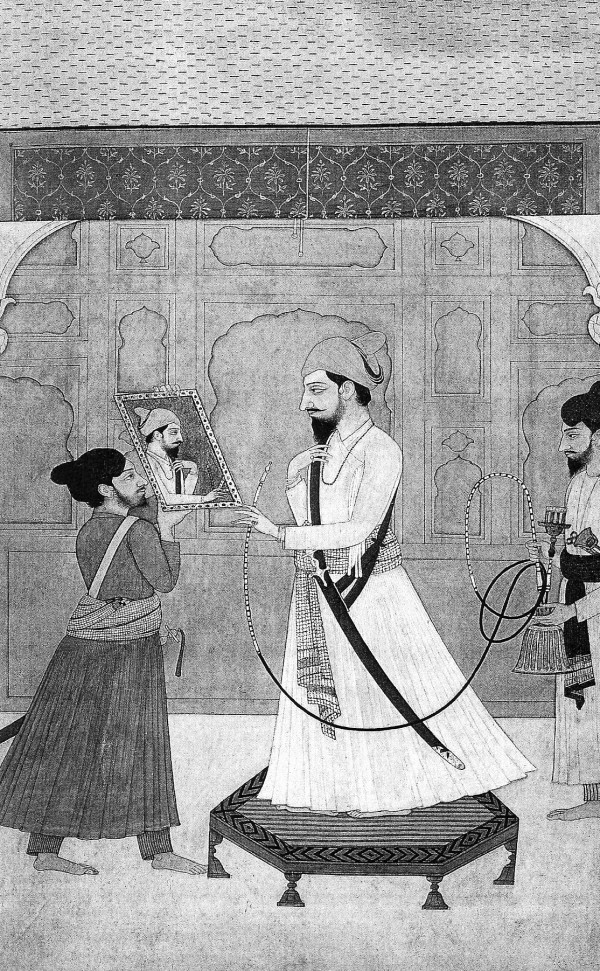
**Raja Prakash Chand**. Ec. Pahari, Guler, ca. 1775. Source: Pouvoir et désir: miniatures indiennes. Dir. Amina Okada. Paris Musees. Ed. Findakly, 2002.

Hookah smoking is an old and deep-rooted tradition in the Indo-Pakistani subcontinent as evidenced by excerpts of historical references given in annex and translated from Urdu into English by Sajid (see also Figure 1). One of them is a booklet written by a British deputy commissioner of the 19^th ^century. This document contains instructions regarding all aspects of the daily life including hookah smoking [1]. Today, hookah (called narghile and shisha in other parts of the world) smoking is considered as a global "threat" and an "epidemic" by public health officials [2,3].

Effects of smoking on human health have been extensively studied worldwide [4]. Tobacco smoke contains over 4800 different chemicals out of which 69 are carcinogens, and several are tumour promoters or co-carcinogens [5]. There is cumulated evidence for a causal association between cigarette smoking and cancers of the nasal cavities and nasal sinuses, lung, oesophagus (adenocarcinoma), stomach, liver, kidney (renal-cell carcinoma), uterine cervix and myeloid leukaemia [6]. Many studies have also shown that the concentration of carcinoembryonic antigen (CEA), known as a marker of malignant transformation and chronic inflammation, is increased in a variety of cancers: *e.g*. carcinoma of pancreas [7]; uterine cancer [8]; cancers of the lung [9] and breast [10]; and among heavy smokers [11,12]. However, It has also been found elevated in some non-malignant tumours such as pleural effusions [13]. In a recent study, Chen et al. report a close correlation of tissue polypeptide specific antigen (TPS), neuron-specific enolase (NSE), carcinoembryonic antigen (CEA) and beta(2)-microglobulin (beta(2)-mG) in serum with lung cancer, histological grades and lymphoid nodule metastasis [14]. Greater-than-normal values of CEA may therefore indicate the presence of cancer.

Pipe and cigar smoking can also cause lung cancer although the risk is not as high as with cigarette smoking [15]. As for other substances than tobacco, Naghibalhossaini et al have reported that CEA levels in opium smokers are affected [16]. In the last decade of the 20^th ^century, interest was developed in studying the effects of smoking at cellular and molecular levels. Ohwada et al. showed that mRNA and protein expression of CEA were increased in the normal lung tissue from smokers compared with non-smokers or ex-smokers. They proved that CEA expression in non-carcinomatous lung parenchymal tissue was the result of smoking and not of the tumour. They also showed that cigarette smoke could induce CEA mRNA expression in foetal lung derived cells. In addition, CEA might play a role in the recruitment of neutrophils of the lower respiratory tract [17]. Kashiwabara et al. suggested that in healthy smokers, high serum CEA levels are related to high neutrophil levels [18]. Most of the studies on tobacco smoking have been done so far on cigarettes, pipe and cigar users. Effect of hookah smoking on CEA levels has been studied for the first time by Sajid et al in 2007 [19]. This last work showed that CEA levels are also increased in cigarette and mixed hookah/cigarettes/bidis smokers although to a lesser extent in the last case. However, there was a need to study exclusive hookah smokers, i.e. those who do not indulge in any other form of tobacco use. The main objective of this study was to measure CEA levels in such exclusive smokers who, every day, use a hookah filled with about 2 "chattaks" (a local weight unit of about 60 g) of a tobacco-molasses mixture, therefore a quantity of about 120 g.

## Methods

### Subjects

**Figure 2:**
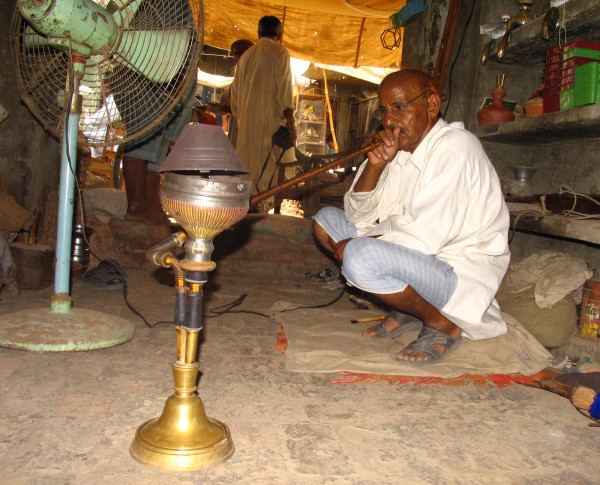
**Traditional Hookah Smoker in Pakistan**. ^© ^Sajid KM. Originally published in Chaouachi K : Tout savoir sur le narguilé; Paris, Maisonneuve et Larose, 2007, 256 pages.

**Figure 3:**
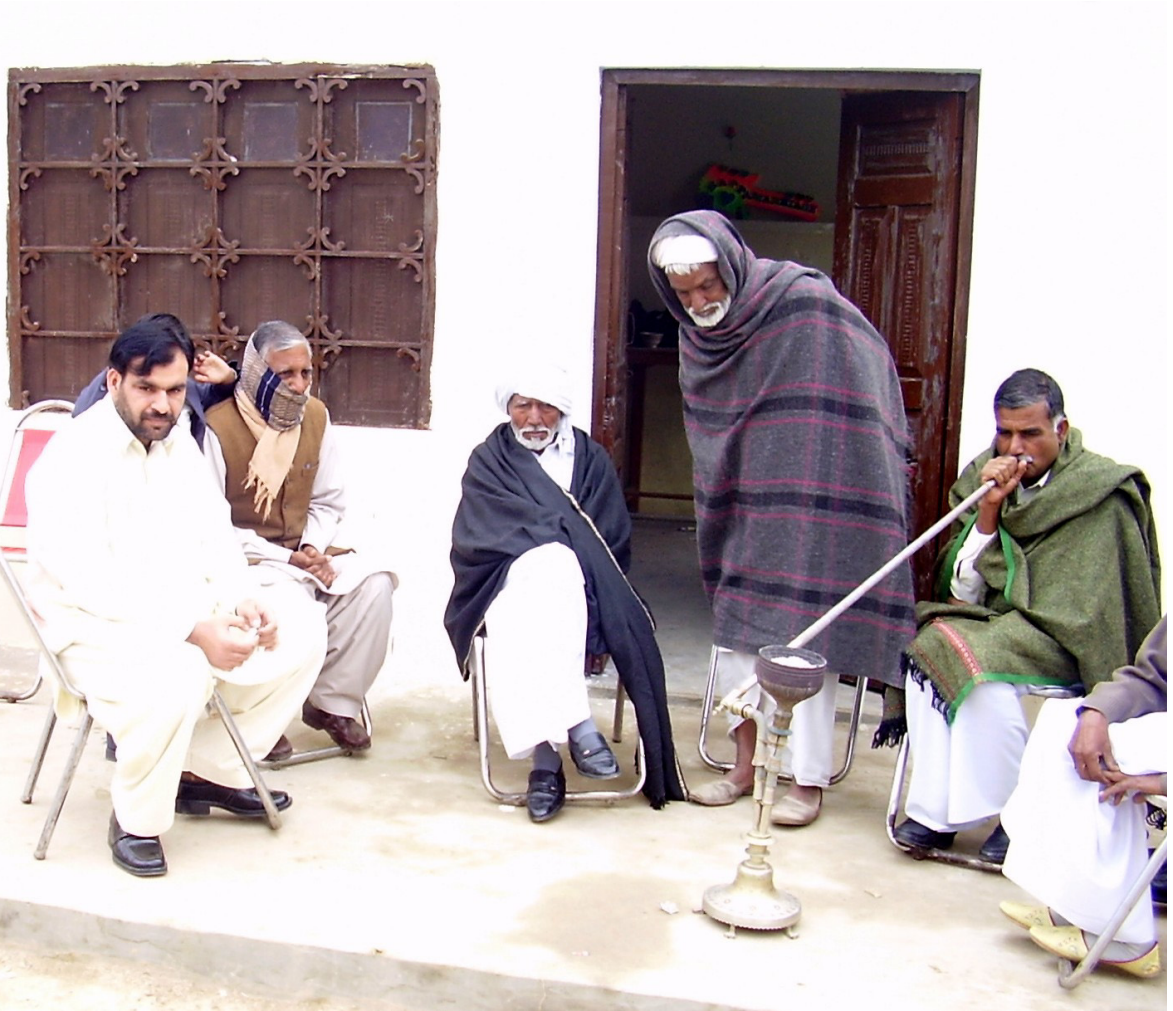
**Traditional Hookah Smoking in Pakistan**. ^© ^Sajid KM.

Volunteers were selected among the farmers belonging to rural areas of Tehsil Burewala (Vehari district) and Jahanian (Khanewal district) in Punjab (Pakistan). In total, 59 male exclusive smokers, with age ranging 20–80 years (mean = 58.8 ± 14.7) and 8–65 years of smoking (mean = 37.7 ± 16.8), participated in this study. The smokers used the hookah in 1–8 daily sessions in the form of 1–4 daily preparations and occasional smokers were excluded. About 1–2 "chattaks" (60 to 120 g; 1 chattak = 58.125 g) of a Desi Punjab tobacco-molasses mixture were used in each preparation. The approximate quantity of tobacco packed inside the chillum (bowl) was almost same in all the subjects. Subjects were divided into 3 groups according to their daily number of preparations, sessions and total smoking time: Light (1; 1; ≤ 20 minutes); Medium (1–3; 1–3; >20 min to ≤ 2 hrs) and Heavy smokers (2–4; 3–8; >2 hrs to ≤ 6 hrs). Before taking blood samples, the subjects were interviewed to make sure that they did not use any other tobacco or smoking product (cigarette, bidis, chewed tobacco). They were clinically examined to exclude any disease (which might influence CEA levels) or narcotic habit and questioned about their smoking career (Figures 2 and 3).

36 non-smokers, who never smoked in their life, served as controls. These controls were hospital staff, university students and individuals from the areas where we collected samples for hookah smokers.

### Materials

**Figure 4:**
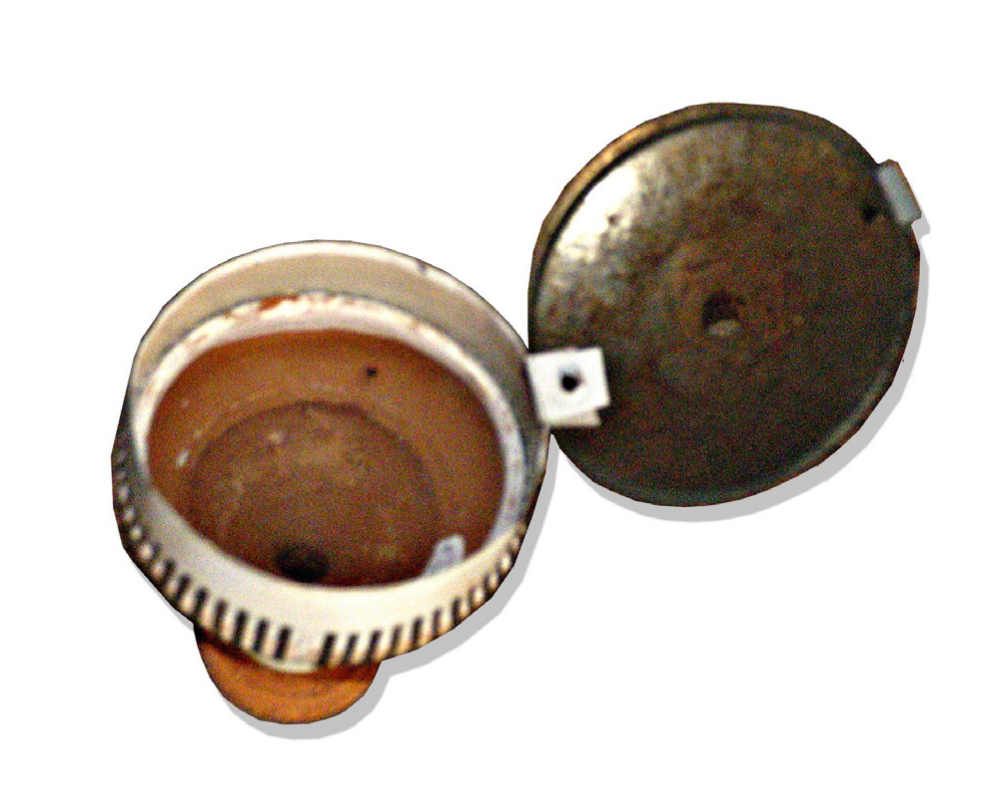
**Hookah Bowl (chilam) from Pakistan**. It is filled with up to 2 chattaks of a tobacco-molasses mixture (i.e. the tobacco weight equivalent of up to 60 cigarettes). ^© ^Sajid KM. Originally published in Chaouachi K : Tout savoir sur le narguilé; Paris, Maisonneuve et Larose, 2007, 256 pages.

**Figure 5:**
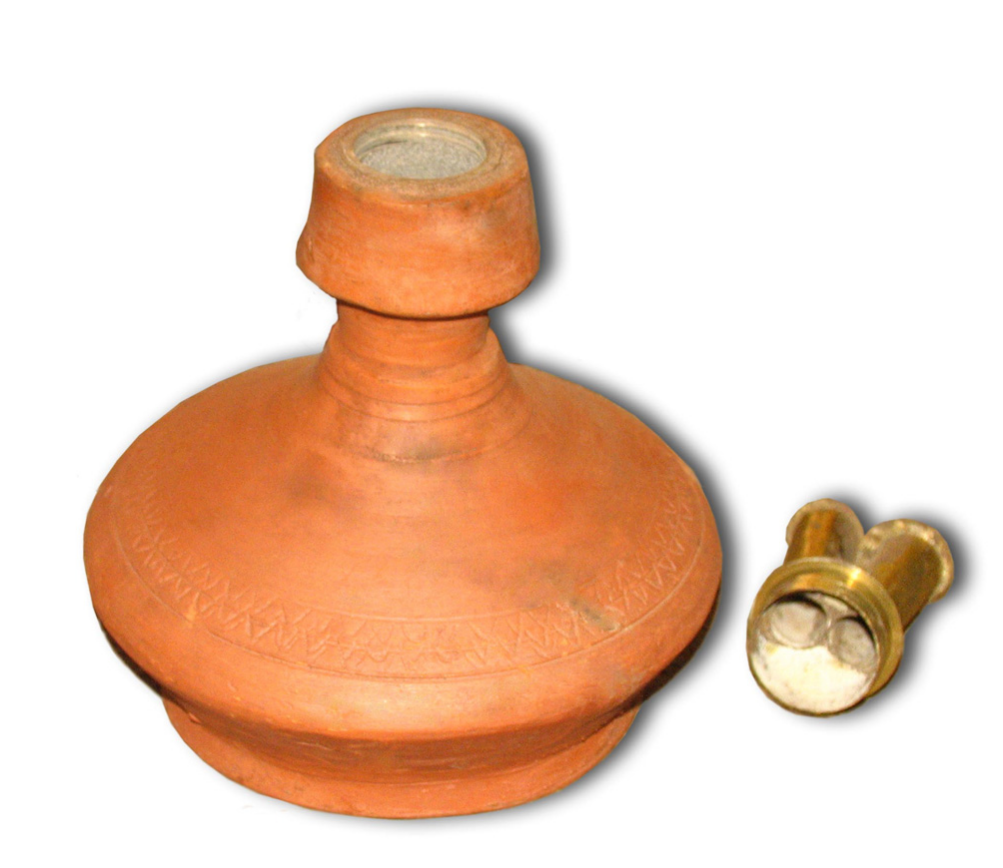
**Hookah Base from Pakistan**. ^© ^Sajid KM. Originally published in Chaouachi K : Tout savoir sur le narguilé; Paris, Maisonneuve et Larose, 2007, 256 pages.

Desi Punjab air-dried and sun-cured tobacco leaves were used to prepare a tobacco-molasses mixture. The molasses element is a dense brownish sap formed during purification of cane sugar (extracted by boiling). The final mixture contains molasses and tobacco in 1:1 ratio by weight [19]. It is similar, to a certain extent, to Arabian jurâk, which contains pulpy fruit apart from tobacco and molasses. Such a mixture is cooked and 15 minutes of its smoking "would provide approximately the same amount of tobacco smoke as one cigarette" [20]. Mixing tobacco with molasses is a very ancient habit. A WHO report dates back "the addition of molasses to burley tobacco in the nineteenth century to create "American" blended tobacco" [21]. However, early health-oriented anthropological research on hookah smoking showed that it is much older and can be traced back to the 17^th ^century thanks to the early relation by an Arab traveller in India [22]. The smoking mixture is deposited above a small stone at the base of the Chilam (also spelled "chillum"), a funnel shaped 500–700 ml capacity container at the top of the hookah (Figure 4). Then, several pieces of glowing charcoal are put above. Notably, the tobacco-molasses mixture is not separated from the charcoal by an aluminium foil as is the case with the moassel (tobamel), the contemporaneous worldwide fashionable aromatic tobacco-molasses product, which also contains glycerol and flavouring essences [3]. Here, the charcoal is directly in contact with the tobacco-molasses mixture. The chilam is connected to a wooden pipe dipped in water in an airtight earthen base containing about 1 litre of water (Figure 5). A curved wooden pipe (Nari) of about 1 to 1.5 metre in length allows the user to inhale the smoke, which has bubbled through the liquid.

### Methods

Carcinoembryonic antigen (CEA; a family of glycoproteins, MW~175000-2000 Daltons) was first identified in 1965 by Gold and Freedman in human colon cancer tissue extracts [23]. It is an important tumour marker for the diagnosis and monitoring of the therapy of diseases like colorectal cancer, lung cancer and ovarian cancer where elevated levels are observed. Levels are also raised in smokers but very few studies correlate CEA levels with smoking type and rate. The enhanced chemiluminescence immunometric technique was applied by using the Immulite-2000 system. This technique was introduced by Babson in 1991 [24]. It has gained worldwide acceptance because of its enhanced sensitivity, specificity and complete automation. The procedure principle involves a solid phase, two-site sequential chemiluminescent immunometric assay with two incubations each of 30 minutes. First incubation involves reaction of CEA with a polyclonal or monoclonal antibody coated on a plastic bead as a solid phase. Alkaline phosphatase-labelled antibody is then added to make an antigen antibody complex. Finally chemiluminescent substrate is added to estimate the reaction product by a luminometer. CEA immulite kits manufactured by DPC, USA were used for this purpose. The bead is housed in a proprietary test unit. This test unit serves as the reaction vessel for the immune reaction, the incubation and washing processes, and the signal development. The equipment used for automatic assay performance and measurement is automated immunoassay analyzer (immulite-2000) a continuous, random access instrument. A bar-code reader identifies tubes and reagent containers. After incubating the sample with the alkaline phosphatase reagent, the liquid reaction mixture in the test unit is rapidly separated from the bead when the bead is washed and the test unit is spun at a high speed on its vertical axis. The entire fluid contents (the sample, excess reagent, and wash solution) are transferred to a coaxial waste chamber, which is integral with the test unit. The bead is left with no residual, substrate. Following washing of the bead, addition of the substrate, a phosphate ester of adamantyl dioxetane, triggers the enzymatic reaction with bound alkaline phosphatase and creates the unstable adamantyl dioxetane anion. Breakdown of this unstable anion creates a prolonged 'glow' of light rather than a 'flash' of light and allows a longer window for numerous readings. The test units now enter a section of the instrument that excludes ambient light and allows for a 10-minute incubation for development of the luminescent signal. Light emission is detected by a photomultiplier tube (PMT). When each tube reaches the read station in front of the photomultiplier tube, photon counts are measured for 1 second through a 2°A neutral density filter, which attenuates the signal by a factor of 100. If counts are below a certain level, the attenuator is automatically removed. This automatic attenuation increases 100-fold the dynamic range of the photon multiplier tube. Counts are measured for 12 consecutive 1-second intervals. After discarding highest and lowest counts for each tube, the average counts per second are converted to analyte concentration by means of the stored calibration curves. The IMMULITE instrument has sufficient storage capacity to save data should any interruption of the communication with the external computer occurs and printed reports are generated for each sample by the system's computer.

The reproducibility data provided by the manufacturer at various concentration levels is reproduced in Table 1. The system has an analytical sensitivity of 0.2 ng/ml and a linearity of dilution up to 1 in 8 [25]. The reproducibility data observed in a cancer patient (quadruplicate sample), a smoker (duplicate) and a non-smoker (duplicate sample) are shown in Table 2. Our results show very little variation among repeated results. Usually in immunoassays, variations less than 10% are acceptable. Therefore, the technique used to measure our samples is rugged.

**Table 1:** Reproducibility data provided by the manufacturer at various concentration levels

Intraassay precision^1,2,3^				Interassay precision^1,2,3^			
	Mean Concentration (ng/ml)	SD	CV		Mean Concentration (ng/ml)	SD	CV

1	1.5	0.08	5.3%	1	3.9	0.26	6.7
2	3.0	0.14	4.7%	2	15	0.84	5.6
3	13	0.47	3.6%	3	60	3.2	5.3
4	56	2.4	4.3%	4	371	22	5.9
5	316	18	5.7%				

1. Beard DB, Haskell CM: Carcinoembryonic antigen in breast cancer. Am J Med 1986 Feb:80:241–245.

2. Begent R: The value of carcinoembryonic antigen measurement in clinical practice. Ann Clin Biochem 1984;21: 231–238.

3. 3. Begent R, Ruslin GJS: Tumour markers: from carcinoembryonic antigen to products of hybridoma technology. Cancer Surv 1989:8(1):107–121.

(Source: manufacturer technical booklet. See SIEMENS in references list)

**Table 2:** Reproducibility data observed in a cancer patient, a smoker and a non-smoker

	**Counts per second (CPS)**	**CEA (ng/ml)**
**Patient with high CEA**		
Replicate-1:	10606939	67.02
Replicate-2:	11254190	71.17
Replicate-3:	10843538	68.50
Replicate-4:	11468146	72.67
Mean ± SD	11043203 ± 389565.76	69.84 ± 2.55
%CV	3.53	3.65

**Smoker**		

Replicate-1:	912044.4	4.82
Replicate-2:	970260	5.23
Replicate-3:	989665	5.3
Mean ± SD	957323 ± 40395.28	5.12 ± 0.259
%CV	4.2	5.06

**Non-smoker**		

Replicate-1:	409945.7	1.603
Replicate-2:	427858	1.718
Replicate-3:	432136.58 (1.01)	1.746
Mean ± SD	423313.3 ± 11772.8	1.689 ± 0.076
%CV	2.78	4.49

5 cc of blood was taken from each smoker. Serum, obtained after clotting and centrifugation, was used in immunoassays for the determination of CEA levels. Samples were stored at 4°C in a fridge for future use.

Details about water changing, hookah size and actually used tobacco amount were precisely recorded during careful interviews with smokers. The protocol of blood sampling, blood handling and assay performance was reviewed by the medical board of Multan Institute of Nuclear Medicine and Radiotherapy (MINAR). It was considered exempt from Pakistani human subjects regulations because the analysis did not expose the volunteers to risk. There were no special ethical considerations in this study because no surgical and other interventions were involved. The safety of subjects was secured. All the subjects voluntarily donated their blood samples. The smokers were exhaustively and previously informed about the significance of the test and were assured of privacy regarding test values. Upon arrival at the local dispensary, participants reviewed study procedures with the medical staff and then completed blood sampling. All data were collected between August and December 2007.

Distribution curves were constructed from the data obtained on non-smokers and smokers (see Results section). Because of the nature of distribution of CEA levels among our individuals (it was not normal), Wilcoxon's rank sum two-sample test was applied to compare the variables using a program provided by the Institute of Phonetic Sciences of the University of Amsterdam (The Netherlands)[26].

## Results

The CEA levels observed in different groups of exclusive hookah smokers are summarized in Table 3 in which data from our previous study (collected according to the same method) on cigarettes and mixed hookah (cigarettes, bidis) smokers are also reproduced [19]. The mean age of the smokers was 58.8 ± 14.7 years. Mean (± SD) levels in light, medium and heavy smokers were: 1.06 ± 0.492 ng/ml (n = 5); 2.52 ± 1.15 ng/ml (n = 28) and 5.11 ± 3.08 ng/ml (n = 26) respectively.

**Table 3:** CEA levels in exclusive hookah smokers

		**Number of daily preparations**	**Number of daily sessions**	**Total daily smoking duration**	**CEA mean levels (ng/ml)**	**p-values Smokers vs non-smokers**
**Light Smokers (n = 5)**		1	1	≤ 20 minutes	1.06 ± 0.492	≤ 0.002157
**Medium **	**Smokers**	1 to 3	1 to 3	≤ 2 hours	2.52 ± 1.15	≤ 0.9138
**(n=28)**
**Heavy **	**Smokers**	2 to 4	3 to 8	≤ 6 hours	5.11 ± 3.08	≤ 0.0001567
**(n=26)**
**Overall hookah **	**exclusive smokers**	1 to 4	1–8	≤ 20 min-6 hrs	3.58 ± 2.61	≤ 0.0937
**(n=59)**
**Non-smokers (n = 36)**		-	-	-	2.35 ± 0.71	-
*Cigarette (n = 122)	**smokers**	-	-	-	9.19 ± 14.9	≤ 5.0 × 10^-7^
*Mixed hookah/cigarette smokers (n = 14)		-	-	-	7.16 ± 10.38	≤ 0.006069

*Data from previous study on cigarettes and mixed hookah (cigarettes, bids) smokers (Sajid et al 2007) aggregated.

**Figure 6:**
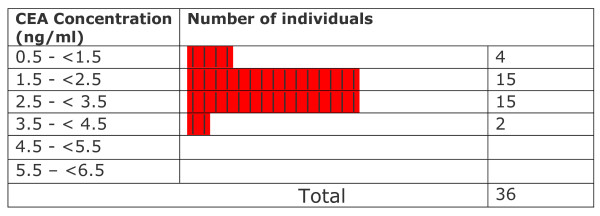
Frequency distribution of non-smokers with respect to CEA levels.

**Figure 7:**
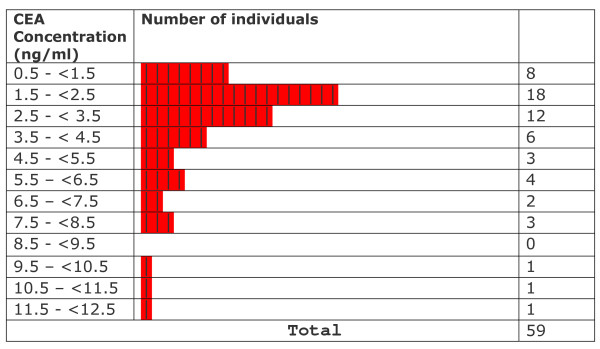
Frequency distribution of all smokers with respect to CEA levels.

**Figure 8:**
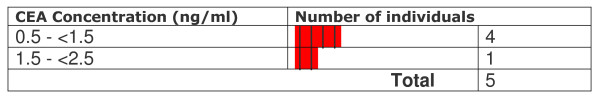
Frequency distribution of light smokers with respect to CEA levels.

**Figure 9:**
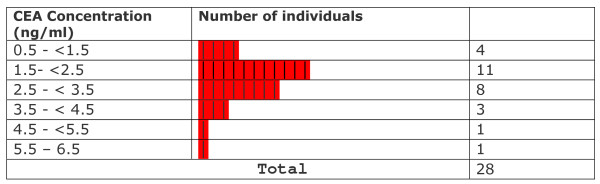
Frequency distribution of medium smokers with respect to CEA levels.

**Figure 10:**
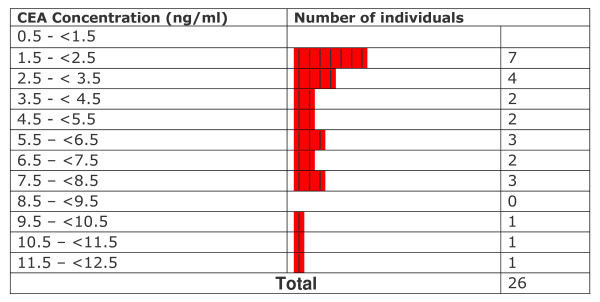
Frequency distribution of heavy smokers with respect to CEA levels.

All CEA levels in light smokers were within normal limits provided by the kit manufacturer. Statistical testing showed that levels in these subjects were significantly lower than the mean levels of non-smokers (p ≤ 0.002157). CEA levels observed in medium smokers were not significantly different from those in non-smokers (p ≤ 0.9138) although at least 6 out of 28 smokers (~21%) crossed the upper normal limit (3.2 ng CEA/ml for non smokers). In heavy smokers, the levels were significantly higher than in non-smokers (p ≤ 0.0001567). A range of values between 0.664–1.90 ng/ml was observed in light smokers (n = 5; % High = 0/5 = 0%). In medium smokers, the range of values was between 0.773 and 5.61 ng/ml (n = 28 ; %High = 6/28 = 21.4%). Among heavy smokers, the range of values was between 1.50 and 12.4 ng/ml (n = 26 %high= 16/26= 61.5%). Levels varied widely and were significantly higher than those observed in non-smokers. Overall values observed in exclusive hookah smokers ranged from 0.664 to 12.4 ng/ml (n = 59 %high = 22/59 = 37.22). The overall mean CEA level in exclusive hookah smokers (3.58 ± 2.61 ng/ml; n = 59) was not significantly different (p ≤ 0.0937) from the level in non-smokers (2.35 ± 0.71 ng/ml). In our first study, cigarette smokers and mixed hookah/cigarette had a mean level of 9.19 ± 14.9 ng/ml (n = 122) and 7.16 ± 10.38 ng/ml (n = 14) respectively [19]. The frequency distribution of data with respect to CEA levels in different groups is displayed in Figures 6, 7, 8, 9, 10. Comparison of these values with non-smokers using Wilcoxon's rank sum test shows that these values are significantly raised compared to values of non-smokers (p-values ≤ 5.0 × 10^-7 ^and ≤ 0.006069 respectively).

## Discussion

### General health aspects of hookah smoking

Health effects of hookah smoking have been regularly reviewed, updated and summarised over the last decade [3,27]. CO levels are generally high, as early established by Sajid et al in 1993 [28]. However, the diverse types of charcoal, tobacco-based mixtures, the size of the device and, above all, the degree of ventilation (indoors, outdoors), play an important role in variations [3,28]. Expired mean CO levels in smokers may thus reach values as low as 14.2 ppm in Jordan [29] and as high as 38.5 ppm at the end of a session (with quick-lighting charcoal) in a Lebanese café where patrons smoked cigarettes and hookah [30]. Levels are even more elevated in some ill-ventilated hookah lounges in France [3]. A study by Al-Kubati et al. on heart rate in hookah smokers, cited in a recent Cochrane review, did not assume that carbon monoxide levels might, perhaps, also account for observed differences with cigarettes, rather than the nicotine itself only [31,32]. Besides, only 5 grams of the smoking product was used for a whole 45 minute session when the common value retained in most reliable studies of the Middle East are 20 grams [29,30]. In these hard experimental conditions, the smoking mixture may char rapidly with a consequent overproduction of CO, carbonaceous material and tar [33,34]. A recent study involving machine smoking shows that hookah mainstream smoke CO concentration is up to 13 times inferior than in cigarettes: 1.79 mg for a 1000 ml hookah (machine) puff and 1.06 mg for a 45 ml cigarette puff [35]. Another area of public health concern in the context of the global epidemic of hookah smoking is ETS (Environmental Tobacco Smoke). There has been a serious debate over statistics about cigarette ETS and their interpretation [36]. However, and in striking contrast with cigarettes, hookah does generate almost no side-stream smoke because of its peculiarities (charcoal topping the bowl and less elevated temperatures). So, the only smoke that should be taken into account is the one rejected by the smoker, *i.e*. the one filtered by the hookah at the level of the bowl, inside the water, along the hose and then by the smoker's lungs themselves. Consequently, the resulting smoke is expected to be less toxic for non-smokers than cigarette side-stream smoke. Notably, a great proportion of irritants, mainly aldehydes and phenols, are removed [19]. A team led by Guillerm in France early found that when passed through water (50 cm3), the combustion gases of cigarette smoke have no inhibitory effect on the respiratory epithelium cilia. The researchers concluded that narghile users can, "without apparent disorders, smoke dramatically greater quantities of tobacco than ours in our countries" [37]. Wynder et al have established that water filtered cigarette smoke is less toxic to clam gill tissue and that "a flask containing 200 ml of water dramatically can reduce the dose of ciliatoxic agents delivered to the ciliated epithelium" [38]. Weiss also reported that the effect of bubbling tobacco smoke through 15 ml of water was "equivalent to the effect of the better charcoal filters" [39]. Zaga and Gattavecchia have shown that the water in the vase of a hookah acts as an antioxydant against some short half-life free radicals [40]. Other substances are supposed to be affected by the water obstacle because of their solubility or the low temperatures: e.g. HCN, nitric oxides, etc. As for particles in the mainstream smoke, and particularly ultra fine ones (0.02 to 1 μm), a recent study shows that hookah smoke is up to 3 times less concentrated than cigarette smoke: 74.4 10^9 ^for a 1000 ml hookah (machine) puff and 9.24 10^9 ^for a 45 ml cigarette "puff" [35]. Similarly to ETS, there is a serious debate on the so-called "nicotine addiction" as this alkaloid is not seen as the central substance involved in the dependence phenomenon [41,42]. Also, it is not the most dangerous one. Hookah dependence is very specific and research on it will help reconsider the "nicotine addiction" hypothesis. Recently, a Lebanese team has found that more than 90% of so-called "mild smokers" (3 pipes or less per week) and about 50% of the so-called "moderate" ones (3 to 6 pipes per week) are considered as non dependent [43]. A certain confusion is also a direct result of the misuse of smoking machines [33,34]. A team in Kuwait has established with a rigorous methodology that the nicotine intake is not as high as in cigarettes [44]. Some of the smokers in our study, particularly the "heavy" ones, were obviously dependent. Further research is needed in this field and it is certainly too early to suggest the use of Nicotine "Replacement" Therapies and products to "hookah addicts", bupropion [32] or even Varenicline produced by Pfizer laboratories and marketed as Chantix and Champix.

### Specific aspects of hookah smoking in Pakistan

Tobacco smoking generally elevates CEA concentrations as reflected in the work by Fukuda et al [12], Alexander et al [11], Naghilbahossaini et al [16], summarised in Table 4. In our study, the subjects were divided into 3 groups according to the number of preparations; the number of sessions and the total daily smoking time: Light (1; 1; ≤ 20 minutes); Medium (1–3; 1–3; >20 min to ≤ 2 hrs) and Heavy smokers (2–4; 3–8; >2 hrs to ≤ 6 hrs). CEA mean level in light smokers (1.06 ± 0.492 ng/ml; n = 5) was significantly low when compared with values in non-smokers (2.35+0.71 ng/ml; n = 36). The low mean level of light smokers relative to the non-smokers does not necessarily indicate that low-level hookah smokers are free from any health risk. Instead of drawing definitive conclusions about these apparently low values relative to non-smokers, we think that the effect of low assay (analytical) sensitivity at lower concentration region, small size of data and some cross interferences in the reaction mixture, should also be taken into consideration. The increased CV in the reproducibility data at (and below of course) 1.6 ng CEA/ml could also be one of the reasons. The mean CEA level in medium smokers (2.52 ± 1.15 ng/ml; n = 28) was not significantly different from the levels in non-smokers. The overall CEA levels (3.58 ± 2.61 ng/ml; n = 59) are thus not affected by the levels in light and medium smokers. However, CEA mean level in heavy smokers (5.11 ± 3.08 ng/ml; n = 26) was significantly raised relative to non-smokers. These values are low compared to those reported in cigarette smokers (9.19 ± 14.9 ng/ml; n = 122) and mixed cigarette/hookah smokers (7.16 ± 10.38 ng/ml; n = 14) in our previous study [19]. This shows that the daily rate of smoking and its duration effects CEA levels. Also, noteworthy is the fact that the quantities of the tobacco mixture involved in hookah smoking in Pakistan are high if compared with cigarettes: up to 120 times (60 times by taking into account the molasses element [19].

**Table 4:** Tobacco smoking and elevation of CEA concentrations

**CEA mean levels (ng/ml)**	**Smokers**	**Non-smokers**
Fukuda et al^12^	3.11 ± 1.8 (n = 467)	2.14 ± 1.8 (n = 874)
Alexander et al^11^	2.7 (n = 154)	1.9 (n = 122)
Naghilbahossaini et al^16^	1.84 ± 0.97 (n = 44). Opium smokers: 3.09 ± 3.00 (n = 128)	1.77 ± 0.86 (n = 47)

CEA mean levels in smokers and non-smokers in the available literature

The range of "normal" values provided by the manufacturer of our kits (DPC, USA) were: 2.1–6.2 ng/ml for male smokers (153 individuals study) and 1.3–4.9 ng/ml for female smokers (81 individuals study). The overall range of values (males+females) in these individuals was 1.3–6.2 ng/ml. The range was 1.1–3.2 ng/ml and 0.8–2.5 ng/ml for male (226 persons study) and female non-smokers respectively (262 persons study). The overall range (males+females) of values in non-smokers was 0.8 – 3.2.

In our last study we observed a mean CEA level of: 9.19 ng/ml (± 14.9) in 122 cigarette smokers; 7.16 ± 10.38 in mixed hookah smokers; and 2.35 ng/ml (± 0.71) in non-smokers [19]. Regarding levels in cigarette smokers, the chemiluminescent immunoassay, more sensitive and specific than the technique (Hansen-Z-gel) used by Alexander et al [11], may account for the differences. However, the origin and quality of tobacco and the environment should also be taken into consideration.

In our last study, CEA levels increased with the number of cigarettes smoked per day and the highest levels were reached by users who smoked more than 31 cigarettes per day [19]. Doll and Peto had established that the annual lung cancer incidence is: 0.273 × 10^-12^. (cigarettes/day+6)^2^.(age-22.5)^4.5 ^[45]. However, some decades ago, people used to smoke at a later age than today. The formula was revisited by Hill who insisted that the most relevant parameter for the assessment of tobacco consumption is duration and not dose (expressed in pack-years) [46]. If consumption is doubled, the risk is multiplied by 2. In striking contrast, if the duration is increased twofold, the risk is multiplied by 23 (2^4.5 ^exactly). Therefore, the exposure duration to tobacco smoke is much more important than the daily number of cigarettes. One conclusion is that quitting as early as possible remains the most powerful factor. Although critical, Lebeau also considers that there is no treshhold dose for risk [47].

In the case of the world fashionable tobacco-molasses smoking mixture called moassel (tobamel), users feel the smoke is very mild (because of the actual water trapping of notorious irritants such as aldehydes) and one direct consequence is that they often inhale considerable amounts of the smoke: randomly varying between 100 ml at least (but less sometimes) and up to 500 ml and sometimes more. These quantities of smoke go directly into their lungs with no previous dilution and stocking inside the mouth (as in the case of cigarettes)[3]. However, in the case of the Desi Punjab tobacco-molasses mixture smoked in Pakistan, and probably because of the different composition (no glycerol, more nicotine) and the higher temperatures, the smoke is not inhaled directly into the lungs and therefore it is diluted. Zahran stated that 15 minutes of its smoking "would provide approximately the same amount of tobacco smoke as one cigarette"[20].

A careful data analysis of our samples shows that CEA values are not normally distributed among the individuals of the various groups (Figures 6, 7, 8, 9, 10). We have therefore applied Wilconson's test to compare CEA values among the different groups. The p-values we obtained this way lead us to almost the same conclusions as those we get to with the t-test except for the comparison between overall CEA levels and non-smokers, where we get a non-significant result in Wilcoxon's test in contrast to a significant one in t-test. This may be due to the high sensitivity of Wilcoxon test over t-test. We are not getting a bimodal pattern in the distribution curve for our smokers, so the grouping according to such a pattern cannot be strictly followed. Therefore, the grouping on the basis of smoking level remains the good criterion for the grouping of our subjects.

The CEA test, although widely used for diagnosis and monitoring the therapy of cancer is not 100% specific for this disease [48]. So when someone's CEA level is high, it does not necessarily mean that the patient has cancer. Similarly, if someone's CEA level is low or close to lower normal limit, it does not mean that the individual is protected from cancer. In the light of these facts, we decided not to merge the light and medium groups of smokers.

### Hookah smoking and Cancer

53 years ago, the British Medical Journal tried to answer the following question: "Does the custom of filtering tobacco smoke through water as in the Eastern hookah remove the noxious elements? Carcinoma of the lung is very rare, in my experience, in hookah-smoking Indians" [49]. A review of what research says about hookah smoking and cancer is therefore necessary to understand the possible influence of the type of exclusive hookah smoking ("light" and "medium" vs. "heavy") on CEA levels, supposed to reflect cancer risks. About half a century ago, Rakower and Fatal investigated this issue further to their analysis of rare available epidemiological statistics on narghile smoking [50]. However, the pioneer in this field is Angel Roffo in 1939 [51]. A recent review of his work was recently published although his very study on narghile was not included for some reason [52]. Roffo was the first to design a machine for the analysis of narghile smoke chemistry. He examined the fluorescence and spectrography of the tar filtered by both a water (narghile) and cotton filter. He was surprised by the filtration rate of the water itself: about 30%. However, he concluded that water and cotton filters could not be used as a means of "absolute prevention" for the prophylaxis of cancer. Lesions on animals appeared more lately than when the tar was directly applied on them. In the researcher's view, this represents a way to reduce the harm of tobacco use ("un medio de aminorar la accion del tabaquismo")[51]. Rakower and Fatal used a more sophisticated smoking machine and speculated on the reasons for which lung cancer would be less prevalent among narghile users: particularly the lower temperatures in relation to the formation of PAH (polynuclear aromatic hydrocarbons)[50]. This study has been sometimes cited in the available literature to support the statement that narghile smoking causes lung cancer [53], i.e. the opposite finding reached by its authors. This has resulted in a wide confusion [54]. More recently, a renowned Syrian lung specialist who has extensively studied narghile smokers, concluded that the low temperatures and filtration of part of the tar may account for the low rates of cancer observed in her country [55].

The hazards of tar, and particularly its carcinogenicity are directly related to the working temperatures whereby not only combustion and pyrolysis are involved, as a WHO report states, but also distillation, as emphasised by Baker et al [21,56]. This is particularly true in the case of shisha smoking where the temperatures of the tobacco-molasses mixture in the bowl does not go in excess of 150°C, allowing a chemical reaction of the Maillard type [27]. Even when using a smoking machine and despite the bias these methods entail, the temperature hardly reaches 200°C [34,35]. The more the temperature is elevated, the more carcinogenic the smoke is. In these conditions, hookah tar is qualitatively very different from that produced by cigarettes. Furthermore, in the case of the fashionable shisha (using flavoured molasses tobacco with glycerol), a great portion of the calculated "tar" is expected to be made up of glycerol which has proved not "adversely alter the smoke chemistry or biological effects normally associated with exposure to mainstream cigarette smoke" [57] as in the harm reduction Eclipse cigarette (about 40%). In any case, what might be really hazardous for tobacco smokers are, apart from PAH, nitrosamines the weight of which does not reach, in certain brands of cigarettes, the 10,000th part of the tar figures printed on the packets. Gray et al, in a study on Polish products, showed that cigarettes containing more nitrosamines were not those with a higher tar content [58]. Consequently, the rating of tar, particularly produced by smoking machines, makes no sense and may deceive tobacco users. Sound and deep research is needed in this field, all the more that a recent study by Sepetdjian et al, based on a smoking machine, has found great amounts of carcinogenic PAH [59]. The underlying methods, including the smoking topography, have been criticised for considerably reducing a highly complex human and social situation [34]. For example, the FTC (Federal Trade Commission) and ISO norms suggest the use of a 1 minute machine smoking interval between 2 puffs in the case of cigarettes for which the duration of a session barely exceeds 5 minutes. However, and by a striking contrast, the hookah smoking device used in the laboratory was based on steady puffs every 17 seconds. This implies that about 1 out of every 4 puffs is supposed to be a human breath for a whole one-hour session (171 regular puffs were drawn this way). Also, contrary to common practice in the real life, the charcoal was left in the same position over the bowl during all this period. In these conditions, the nature and yields of toxicants in the smoke are questionable. Furthermore, the low temperatures involved, as highlighted several times in our study [27,33-35,50,55], do not theoretically allow for the abundant formation of hazardous PAHs. Sepetdjian et al suggest that one source for the PAHs might be the charcoal and that different types of the latter might induce different yields. As in the case of CO, Sajid et al had early established in 1993 that concentrations of this gas depend on the nature of charcoal (natural vs. commercial)[28]. In any case, assays on human subjects (urinary carcinogens, chemical or biological markers) would be more appropriate as in our previous study and the present one [19].

## Conclusion

As far back as 1965, Wynder and Hoffmann concluded their historic proposal for tobacco research programs as follows: "The best way to avoid the risk of those types of cancer associated with tobacco use, and particularly with cigarettes smoking, is to stop smoking entirely. In view of the fact that man may not always accomplish this objective, research efforts towards reducing the experimentally established tumorigenicity of smoking products should be vigorously continued"[60]. This is true for hookah smoking and our study shows that, as far as CEA levels are concerned, heavy smokers (spending up to 6 hours per day in 3 to 8 smoking sessions of a tobacco weight equivalent to about 60 cigarettes) are very much at risk than the medium smokers (up to 2 hrs per day in 1 to 3 smoking sessions) or the light ones (up to 20 min per day in 1 smoking session). If traditional hookah smoking, as exemplified by the Pakistani context, has fewer carcinogenic effects than cigarette smoking, it is important to bear in mind that it still produces smoke. What is hazardous with tobacco use is the smoke, not the tobacco itself. In other words, smoked tobacco is much more dangerous than tobacco used in other forms as renowned scholars have early warned [61,62].

However, it must be clear that there are important differences between smokeless products. On one hand, some of them, like the Sudanese "tumbak", contains high levels of carcinogenic nitrosamines [63] and we fear that the Pakistani "naswar" might be of a similar nature. There is dramatic dearth of research in this field. On the other hand, a moist snuff like the Swedish SNUS, is, in the view of prominent international experts, highly recommendable all the more that it is also very low in carcinogenic substances [62,64,65]. In a broad harm reduction perspective, we therefore encourage the use of smokeless tobacco of the Swedish SNUS type. Heavy hookahs users, cigarette smokers and users of unknown quality local snuff should be invited to consider the health benefits of switching to this new product. Such a harm reduction product is culturally adapted to the Pakistani context and other similarly sanitary and socio-cultural ones.

## Competing interests

The authors declare that they have no competing financial interests and that they have never received direct or indirect funding neither from pharmaceutical companies nor from the tobacco industry. Dr Kamal Chaouachi was involved from Spring to Autumn 2004 in the development of a no-carbon monoxide narghile prototype (harm reduction objective). He signed away all his past and future rights (total relinquishment, including rights related to a patent he was a co-author of) on June 15, 2005 (legally certified by State Attorney in Paris), before the potential commercial exploitation of the product. He has received only a lump sum for his participation in this project. Furthermore, he declares that, in the course of his 10-year research work on this issue, he has never received direct or indirect funding neither from pharmaceutical companies nor from the tobacco industry.

## Authors' contributions

KMS developed the basic idea of the study, carried out the experiments, worked on the theoretical aspects, participated in data analysis and in the writing of the manuscript. His overall contribution was 35%. KC developed the basic idea of the study, worked on the theoretical aspects, participated in data analysis and in the writing of the manuscript. His overall contribution was 35%. RM carried out the experiments, participated in data analysis and in the writing of the manuscript. Her overall contribution was 30%. All authors have read and approved the final version of the manuscript.

## Annex 1

### Diary of a Deputy Commissioner (DC)

In 1889 the British Deputy Commissioner (DC; Chief Controlling Officer of a district; mostly British national) in colonial India used to follow a small booklet written by an English DC of British times. The booklet contained instructions, which were compulsory for each DC to follow in order to control and frighten the public of their area. The instructions regarding number and kind of their personal staff were interesting. For each DC it was essential to keep following staff for his personal service at the expense of government.

Bera (table servant):1, Butler:1, Khansama (Cook):1–3, Servant:1, Sag Bardar (Dog carer):1, Saees (Horse carer):2, Massalchi (Massager), Hammal (porter; to carry the load):1, House maid:1, Hookah Bardar (hookah servant):1, Dhobi (Washerman):1, Darzi(tailor):1, Bahishti (Water carrier/sprinkler):1, Mali (gardener):1, Nai (Barber):1, Doodh wala (Milkman):1, Mehtar(sweeper):1, Pankha Quli (Fan operator);1, Patta Dar (Peons):3.

Queen used to rule India in those days. The empire was so big in those days that the sun did not set in the area occupied by the Great Britain. The D.Cs used to drink boiled water and eat the fruits dipped in antiseptic solutions. In order to avoid hot air they used to wear falalain (Flannel; special kind of cloth with soft and thin fiber) and used hand gloves and long shoes to avoid the mosquitoes.

In the brigade of their servants, hookah servant had a special rank. The cigarettes and cigars were not common in those days. However pipes were common but where East India Company left India for queen it also transferred hierarchy of hookah. Hookah smoking was very common in the beginning of 20^th ^century. English officers used to keep very decorated and attractive hookahs. The water used in these hookahs was mixed with flower extracts having very pleasant smell and the officers used to smoke in a retiring phase (laid on bed or seat of his vehicle). A servant used to handle the pipe of hookah during this period. He also used to blow the burning coal through a copper pipe (phoonkni). All officers used to keep these hookahs with them in lunch and dinner parties. The hookah carriers also accompanied him. After each meal of such parties a procession of hookah carriers used to come to the party venue-carrying hookahs to the party place. Every hookah holder used to take his hookah to his lord and used to stay respectfully for indefinite periods. The hookah sittings were full of manners and to pass over one man's pipe of hookah to the other was thought to be a serious disrespect. The maims (madams; respected ladies; wives of officers) had also the habit of smoking hookah. They used to wave/wrap the long pipes of hookah around their waists like snakes and thus enjoyed smoking. Ilaichi (cardamom; a special kind of seed in India with pleasant smell; used in herbal medicine), saffron and gold leaves (very thin sheets of gold) were mixed with tobacco. The mothers of these maims used to tell very proudly to their neighbors in Europe saying "Our daughters play with snakes and inhale the gold. When the wife of a deputy commissioner wanted to honor someone she allowed him/her to take few puffs of her hookah. On the other hand wife of a session judge wanted to compete her and exhibited her hookah frequently.

## Annex 2

### Other historical references, including translation from Urdu into English by KM Sajid

The Eighteenth century poetry reveals that most of the Indian high class liked hookah the most. The barons used to appoint poets to please them through their lyric creations. Nasikh was one of them. He says when admiring his lord:

Hookah jo hay Huzoor-e-Mualla kay hath mein

Goya keh kehkshan hay surrayya ke hath mein

Yeh sab baja hay laikin ae Nasikh tu arz kar

*Bay jan bolta hay masiha kay hath mein*.

#### Translation

Hookah in the hands of his excellence looks like a galaxy in the hands of Suurayya (a group of seven stars in the sky). That all is true but, O Nasikh, you submit before your lord that hookah [although a non living thing like a dead body] has now become a living thing [person] in the hands of Christ and is now talking to my lord [the hookah gurgling sounding like talking].

Hookah is also a symbol of love or hatred in local poetry (Punjabi). For example, a lover would say:

Hookah Hukm Khuda da chilum hookay di dhee

Jithay pia daikhiyye othay laiyye pee

#### Translation

Hookah is a command of God and chilam (the hookah bowl) is its daughter. Wherever you see it, drink it immediately.

Whereas, a hookah opponent would claim:

Hookah hukm shaitan da chilum hookay di run

Jithay pia daikhiye othay daiyye bhun

#### Translation

Hookah is the command of Saitan (the Devil) and chilam is its wife. Wherever you see it, break it immediately.

Finally, it is noteworthy that for Sikhs of Punjab (Indian side), hookah smoking is religiously banned just like pork is for Muslims and Jews.

## Annex 3

Dhuein da Tharki (The Addict of Smoke). A Poem in Punjabi on Hookah and Cigarette Smoking

This poem was written in its orginal language, and translated into English by Khan M. Sajid himself, on the occasion of a recent tragic event: the death of his brother-in-law, aged 35 years. Its content has a straightforward relation with the issues raised in the Harm Reduction Journal and, also, Kamal Chaouachi’s family misfortune.    

Dr Sajid used to say: “Poetry is nothing but the effect of environment on our minds”. He has written many Haikus (Japanese style)(*) in Punjabi and Urdu and, not the least, six books on poetry. Three of them (in Urdu) have been published and a fourth one has been composed.   

**DHUEIN DA THARKI ** (The Addict of Smoke) 

Budday khangar babay wangun huqqa bur bur bolay

(Hookah is repeatedly sounding "bur bur" like an old man with chronic cough)    

Koray ghut dhuain day bhar kay raz dilan dy kholay

(Taking bitter puffs of hookah smoke reveals the deep hidden secrets of heart)

Kash sigrat da bhar kay jehra apna ap tatolay

(If a person takes a puff of cigarette smoke and then examines his inside) 

Ohi wikahay larday hoay cheelan nal mamolay 

(It is he and only he who observes wagtails fighting with cheels [a bird of prey like falcon])

Pee kay sigrat podder wala waikho ajabanzara

(Smoke a cigarette containing powder [of heroin] and see a strange scene around) 

Parbat di shahzadi aaii beh kay uran khatolay

(The princess of mountains is coming seated in a [legendary] flying car)

Doctor akhan cancer lagda huqqay sigrat kolon

(The doctors say hookah and cigarette cause cancer) 

Mainun tay fun paray lagday sigrat day margholay

(But to me the spirals of smoke look like beautiful pieces of abstract art)

Waikho yaro dais asada kinna aggay wadya

(Oh people! See how much our country has advanced) 

Ik nukkar tay raket baitha peeway sigrat polay

(A "rocket" is sitting on the corner of a street and smoking very soft [“light”] cigarettes)(**)

Chacha Huqqay wala Akhay “Huqqa Hukam Khuda da”

(Our uncle who sells hookahs says: "Hookah is the command of God”)

Aakkho ja kay ohnun loko ainan kufranah tolay

(Ask him to be careful as such kind of words are nothing but infidelity)

Notes (by KM Sajid):

(*) Haiku is actually similar to "Mahya" poetry. However, its is even older and it means "beloved". The only difference between the two is that Mahya uses rhyming words in the first and last lines. See, for instance, one Mahya in the Urdu language [Sajid]:

Hai Kaga Banerei par

Sooraj koii chamkawo

Iss man kai andherie par

Translation: *There is a crow sitting on the top of the wall of my house (sitting of a crow on the wall of a home is thought as a news of arrival of some guest and is therefore a symbol of hope). So please enlighten a sun in the darkness of my mind.*

(**) A heroin addict is called a **"rocket"** among common people. When he is intoxicated, he waves and thinks himself as flying like a rocket and exploring the space. Pola, or soft cigarette, is prepared by the heroin addict. He takes out the tobacco of a cigarette, mixes it with heroin powder, heats it gently and then refills it. This makes the cigarette soft relative to one without heroin powder (effect of manual packing).

**"Light"** certainly means having less weight. “Pola” is used to indicate looseness or slight hollowness due to cohesion of tobacco heroin particles. The weight of a cigarette with heroin is actually increased .
